# Early‐life exposures and child health outcomes: A narrative review of LSN21 research in Japan

**DOI:** 10.1111/ped.70258

**Published:** 2025-11-10

**Authors:** Naomi Matsumoto, Rumi Matsuo, Yuka Yamamura, Takahiro Tsuge, Tomoka Kadowaki, Kensuke Uraguchi, Kei Tamai, Kazue Nakamura, Akihito Takeuchi, Takashi Yorifuji

**Affiliations:** ^1^ Department of Epidemiology, Faculty of Medicine Dentistry and Pharmaceutical Sciences, Okayama University Okayama Japan; ^2^ Department of Epidemiology Okayama University Graduate School of Medicine, Dentistry and Pharmaceutical Sciences Okayama Japan; ^3^ Department of Rehabilitation Kurashiki Medical Center Kurashiki Japan; ^4^ Center for Public Health Action in Applied Epidemiology National Institute of Infectious Diseases, Japan Institute for Health Security Tokyo Japan; ^5^ Department of Otolaryngology‐Head & Neck Surgery Kagawa Rosai Hospital Kagawa Japan; ^6^ Division of Neonatology NHO Okayama Medical Center Okayama Japan; ^7^ Okayama City Public Health Center Okayama Japan

**Keywords:** breastfeeding, child health, environmental exposure, longitudinal studies, perinatal

## Abstract

**Background:**

The Longitudinal Survey of Newborns in the 21st Century (LSN21) tracks two Japanese national birth cohorts—2001 (baseline *n* = 47,010) and 2010 (*n* = 38,554)—from infancy through young adulthood, capturing parenting practices and family environments. Most studies analyze single exposures or outcomes. We conducted a narrative review summarizing the findings published by the Okayama University group on diverse health and developmental outcomes.

**Methods:**

We reviewed 59 LSN21 papers (2013–2025), extracting data on exposures, outcomes, and methods. Evidence was categorized into four exposure types (infant feeding, sleep, environmental, and perinatal) and three outcome domains (obesity, allergies/respiratory tract infections, and neurobehavioral development), including cohort comparisons.

**Results:**

Exclusive breastfeeding was associated with a lower obesity risk at ages 7 (adjusted odds ratio 0.55, 95% confidence interval 0.39–0.78) and 15, later puberty, and fewer hospitalizations. Short or irregular sleep before age 3 was linked to behavioral problems and injuries. Maternal smoking and prenatal air pollution were associated with respiratory conditions and developmental challenges. Preterm birth and small‐for‐gestational‐age predicted delays, especially without catch‐up growth by age 2. Pneumococcal vaccination likely contributed to declining otitis media after 2010. Additional findings included associations between outdoor play and reduced obesity risk, and complex relationships between breastfeeding and food allergies that varied by infantile eczema status.

**Conclusions:**

LSN21 findings highlight modifiable early‐life factors (breastfeeding, sleep patterns, and smoke‐free environments) and identify preterm and growth‐restricted children for priority monitoring. While LSN21's strength lies in longitudinal social assessments, complementary perspectives from other Japanese cohorts could enhance understanding of biological mechanisms and intergenerational effects.

## INTRODUCTION

The past decades have witnessed significant demographic shifts in Japan, characterized by declining birth rates, an aging population, and evolving social structures. These changes prompted the Ministry of Health, Labour and Welfare (MHLW) of Japan to initiate the Longitudinal Survey of Newborns in the 21st Century (LSN21) in 2001,^1^ with a second cohort added in 2010,^2^ to systematically track children from birth through adulthood. This government‐led survey was specifically designed to inform evidence‐based policymaking that addressed declining birth rates and supported child development by collecting multidimensional data on health trajectories, developmental milestones, family dynamics, educational experiences, and socioeconomic factors.^3^

Initially administered solely by the MHLW, the survey transitioned to joint management with the Ministry of Education, Culture, Sports, Science and Technology (MEXT) in 2017, expanding its scope to include educational outcomes and future employment aspects.^1^ The 2010 cohort was deliberately designed to enable direct comparisons with the 2001 cohort, allowing researchers to evaluate generational changes and policy effectiveness over time. This long‐running longitudinal approach has systematically documented growth and developmental patterns in Japanese children through consistent follow‐up assessments, which has generated substantial contributions to maternal‐child health, education, and social welfare policies.

The accumulated evidence has been disseminated through numerous international peer‐reviewed publications. Recent analyses by other institutions have capitalized on LSN21 data—linking household socioeconomic status to Kawasaki disease,^4^ early daycare attendance to reduced preschool wheeze,^5^ and noting widening inequalities in dental caries treatment over early childhood^6^—and demonstrated the versatility of the cohort. However, each of these papers addresses only a single exposure–outcome pair, and an integrated overview across developmental, behavioral, and environmental domains is still lacking.

To address this gap, we present an invited narrative review of 59 peer‐reviewed studies that were based on LSN21 data, conducted by our Okayama University group (Table S1). This overview has two aims. First, by organizing our findings by topic, we provide a coherent picture of child health and development from birth through adolescence and indicate how family and social determinants are associated with child health. Second, we summarize study designs and results, note methodological limitations, and outline broad directions for future research and policy.

The summary presented here is intended as a resource for pediatric clinicians, public health researchers, and policymakers who seek evidence derived from a large, nationally representative birth cohort. By drawing together multiple developmental domains across different ages, we aimed to clarify the complex interplay of early‐life factors and long‐term health outcomes in the Japanese context, insights that may also be relevant to other industrialized countries facing similar demographic challenges.

## METHODS

### Study selection

We reviewed 59 international peer‐reviewed papers, including original articles, research letters, and short communications, based on LSN21 data that were conducted by our Okayama University group and published between January 2013 and April 2025 (Table S1). These papers, identified from our group's internal publication records, were categorized by exposure and outcome domains in Table 1. As this is a narrative review summarizing our group's contributions, no systematic literature search protocol with predefined inclusion or exclusion criteria was applied to studies from other institutions. To note, because we did not perform a systematic search of external literature, the review should be interpreted as a descriptive summary of our group's contributions rather than an exhaustive appraisal of all LSN21‐based research.

**TABLE 1:** Distribution of 59 publications from the Longitudinal Survey of Newborns in the 21st Century (2013–2025) by exposure domains and child health outcome categories.

Exposure domain	Outcome domain			
	Allergic/respiratory	Neurobehavioral	Obesity/growth trajectory	Other outcomes
Infant feeding	Yamakawa 2014 (S6); Yamakawa 2015 (S8); Yorifuji 2016 (S14); Matsumoto 2020 (S30)	Yorifuji 2014 (S4)	Yamakawa 2013 (S2); Kadowaki 2023 (S42); Higuchi 2023 (S43)	Kato 2015 (S9); Nakamura 2020 (S31)
Sleep	Uraguchi 2025 (S53)	Kobayashi 2014 (S5); Kato 2018 (S24); Yamauchi 2022 (S40)		Obara 2021 (S36)
Environmental	Yamakawa 2017 (S17); Yorifuji 2018 (S22); Tokinobu 2018 (S25); Yorifuji 2019 (S28); Uraguchi 2023 (S44); Uraguchi 2023 (S47); Shigehara 2025 (S56)	Yorifuji 2016 (S10); Inoue 2016 (S15); Yorifuji 2017 (S18); Ariyoshi 2022 (S39)	Matsumoto 2021 (S35); Yamashita 2023 (S41); Tsuge 2025 (S57)	Yorifuji 2015 (S7)
Perinatal	Takeuchi 2022 (S37); Takanaga 2024 (S48); Yabuuchi 2025 (S59); Matsumoto 2025 (S55); Matsumoto 2024 (S51)	Kato 2013 (S3); HigaDiez 2016 (S11); Takeuchi 2016 (S12); Takeuchi 2017 (S16); Takeuchi 2018 (S21); Tamai 2019 (S27); Takeuchi 2019 (S29); Tamai 2020 (S32)	Matsumoto 2021 (S35)	Kato 2013 (S1); Nosaka 2016 (S13); Nosaka 2017 (S19); Nakahara 2018 (S20); Yoshimoto 2019 (S26); Tamai 2022 (S38); Ohyama 2023 (S46); Hiraoka 2024 (S52)
Other exposures	Kikkawa 2018 (S13); Matsumoto 2021 (S34); Namba 2023 (S45); Uraguchi 2024 (S49); Kobayashi 2025 (S54); Matsumoto 2025 (S58)	Matsuo 2021 (S33); Murata 2024 (S50)		Nosaka 2016 (S13)

*Note*: The numbers in parentheses (S#) correspond to the listing order in Table S1 and are independent of the reference numbers cited in the main text.

### Sample characteristics and representation

The 2001 Birth Cohort targeted all live‐born infants during 10–17 January, and 10–17 July, 2001, encompassing approximately 54,000 children in the baseline assessment. The initial response rate was 87.7%, with proportional representation from both birth months (January: 23,421 children, 88.0%; July: 23,589 children, 87.5%). Notably, multiple births (i.e., twins and triplets) were included, with each child registered and tracked individually, ensuring comprehensive representation of all births.^1^ The 2010 Birth Cohort targeted all live‐born infants during 10–24 May, 2010, with approximately 44,000 newborns included, achieving an initial response rate of 88.1%.^2^ Both cohorts covered all 47 Japanese prefectures, with geographical distribution proportional to regional birth rates, thus providing near‐national coverage across urban and rural areas.^7^ Because the time frame is limited to specific birth weeks, strict population representativeness—especially for seasonality—cannot be assumed.

### Survey timeline and methodology

Baseline assessments were conducted at approximately 6‐month postpartum, followed by structured follow‐up surveys. For the 2001 cohort, the first six surveys (at 0.5, 1.5, 2.5, 3.5, 4.5, and 5.5 years) were conducted annually, with January‐born children surveyed each August and July‐born children each February. After the 5.5‐year assessment, the schedule was adjusted to align with school entry, with the seventh survey conducted at age 7 (first grade), followed by annual assessments thereafter. The 2010 cohort utilized a similar approach. The 2001 cohort has been followed for over 20 years, with the 22nd survey conducted in 2022 when participants reached age 21. The 2010 cohort completed its 14th survey in 2024 when participants were 14 years old (second year of junior high school). Both cohorts are still being followed, and the government plans to track participants through key life transitions including higher education, employment, and family formation. Its long follow‐up period makes LSN21 a valuable resource for understanding how early‐life experiences and environments shape developmental pathways into adulthood in the Japanese context. The LSN21 represents one of Japan's most sustained longitudinal studies of child development.

### Data collection domains

Data collection relied on postal self‐administered questionnaires distributed by the MHLW (later, from 2017, co‐administered with the MEXT). For both the 2001 and 2010 birth cohorts, the respondent was a parent or guardian for Waves 1–10 (ages 0–10); starting with Wave 11 (age 11), the target child also completed a self‐administered section. Waves 11–12 combined the child pages with the parent booklet, while from Wave 13 onward, the survey has been split into separate “child” and “parent” booklets for both cohorts. Since 2020, an electronic response option has supplemented the paper‐based format. The follow‐up protocol has maintained high retention rates, with approximately 85% of the original 2010 cohort sample still participating by the ninth survey. Households that miss two consecutive waves are considered attrited and are no longer contacted. The survey applies standard quality‐control procedures specified by the MHLW.^8^

Each wave features age‐appropriate metrics that address multiple domains: physical growth parameters, developmental indices, health status indicators (illnesses, injuries, and hospitalizations), familial environment characteristics, parental attributes (education, employment, and income), educational milestones, childcare utilization patterns, and caregiving attitudes. The 2010 cohort added items regarding the potential demand for childcare services when they are not currently being used and changes in work patterns after childbirth to address evolving social concerns. The survey also captures parental involvement in childcare (particularly fathers' participation), disciplinary approaches, parental stress levels, and access to support networks. As children matured—and from Wave 11 began providing part of the information themselves—questions about school experiences, academic performance, and future aspirations were incorporated.

### Ethical governance

Ethical governance for the primary data collection adheres to the Japanese Statistics Act framework, under which the MHLW ensures compliance with national research standards for government‐led surveys. Participants received comprehensive information about study objectives and confidentiality protocols. Participation remained voluntary throughout all waves, with safeguards implemented for data security and participant anonymity.

Furthermore, regarding the primary data collection, starting with Wave 11 (age 11), children also completed a self‐administered section of the questionnaire; their participation was contingent on parental consent and the child's own willingness to respond, which served as a form of informed assent.

Building on this ethical governance framework, the MHLW publishes summary statistics from each survey wave as “Overview of Results.” For research purposes, anonymized individual‐level data can be accessed through formal application under the Statistics Act. These access procedures balance research utility with participant confidentiality while enabling academic studies across disciplines, including pediatrics, public health, sociology, education, and economics.

All studies included in this review were secondary analyses of these anonymized data and were conducted after receiving approval from the Institutional Review Board of Okayama University Graduate School of Medicine, Dentistry, and Pharmaceutical Sciences (No. 1506‐073). In accordance with our IRB‐approved protocol for using existing data, informed consent for our research was obtained in the form of an opt‐out opportunity provided on a website.

## FINDINGS FROM THE LSN21

### Part 1: Early‐life exposures and health outcomes

#### Breastfeeding

The LSN21 has generated substantial evidence across multiple exposure domains that influence child health and development. Among these, infant feeding practices represent one of the most fundamental early‐life exposures with far‐reaching implications. Nutrition during early infancy significantly impacts the subsequent health and development of children. In particular, breastfeeding is crucial for short‐ and long‐term medical and neurodevelopment outcomes for infants.^9^, ^10^, ^11^, ^12^ However, scientific evidence on the effects of breastfeeding in high‐income countries remains limited,^13^ highlighting the value of the LSN21's longitudinal approach to this topic.

Infant feeding practices in the LSN21 were categorized as “exclusively breastfed,” “partially breastfed,” and “formula‐fed.” For formula‐fed infants, we also considered whether infants had taken colostrum, depending on the purpose of the study. Additionally, “partially breastfed” was divided into three categories (1–2, 3–5, and 6–7 months) according to breastfeeding duration.

**FIGURE 1:**
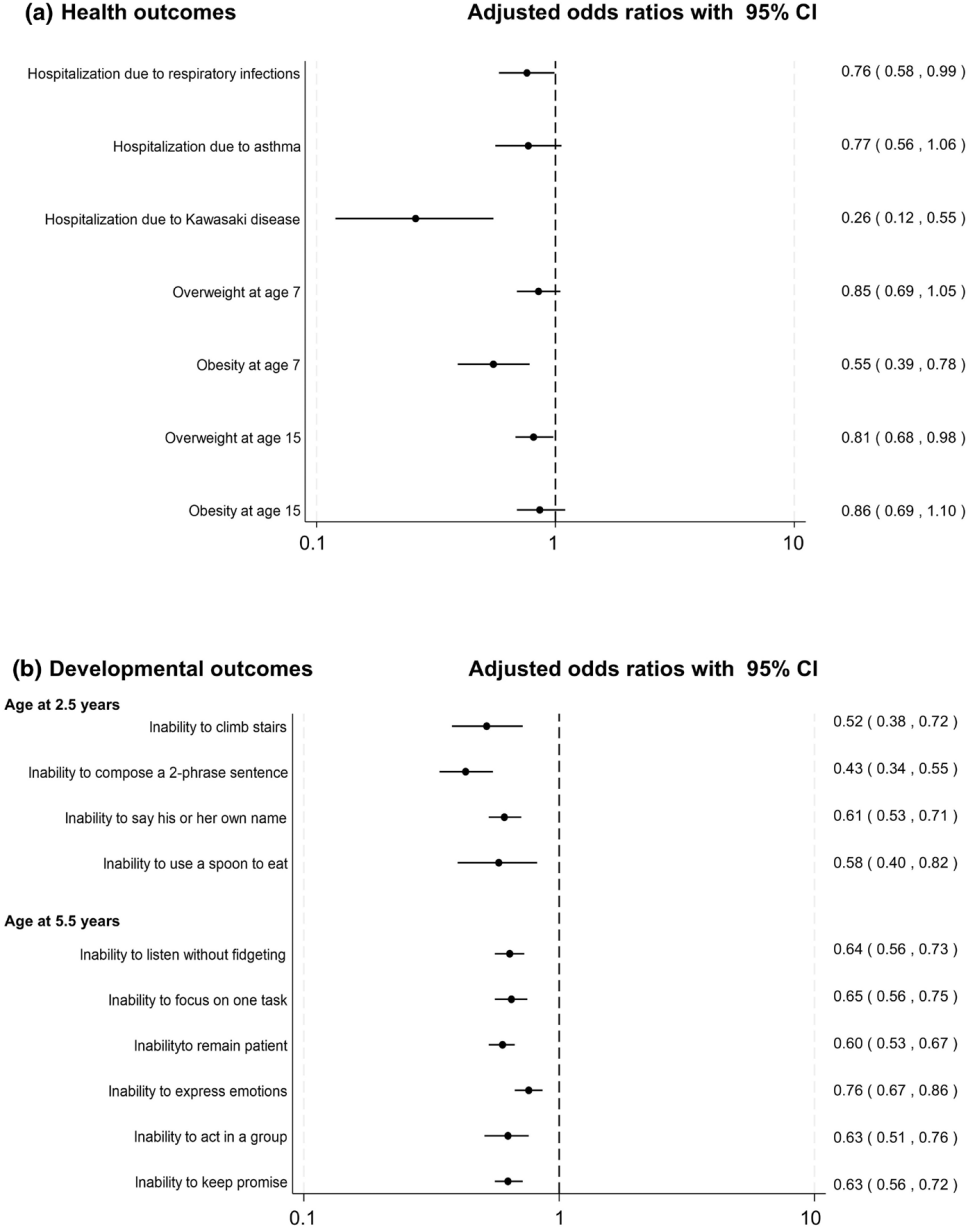
Association of exclusive breastfeeding during the first 6 months and subsequent childhood outcomes in the LSN21 cohort. Panel A. Child health outcomes. The analysis of hospitalization owing to respiratory infection is restricted to term infants only (gestational age ≥37 weeks). Panel B. Developmental outcomes assessed at ages 2.5 and 5.5 years. Dots indicate adjusted odds ratios (ORs) with 95% confidence intervals (CIs) comparing children who were exclusively breastfed with those who received formula only (reference group). For obesity at age 15, the OR was inverted (1/OR) to maintain consistent presentation, as formula‐fed children were used as the reference group in the original analysis. All estimates were adjusted for relevant maternal, child, and socioeconomic characteristics. The x‐axis is presented on a logarithmic scale. Estimates correspond to the following studies from Table S1: Hospitalization due to respiratory infections (S8), hospitalization due to asthma (S6), hospitalization due to Kawasaki disease (S14), overweight and obesity at age 7 (S2), overweight and obesity at age 15 (S42), and developmental outcomes (S4).

Our studies suggested that breastfeeding was associated with multiple aspects of child development and health status (Figure 1a,b). After adjusting for potential confounders, we found that exclusively breastfeeding was associated with lower odds of overweight and obesity through childhood and adolescence,^14^, ^15^ with detailed findings presented in the obesity section. Additionally, breastfed children showed later puberty onset (measured by age at peak height velocity: APV) than formula‐fed counterparts, particularly among girls; for example, the age at peak height velocity for exclusively breastfed girls was significantly later than for formula‐fed girls (standardized regression coefficient *β*: 0.150, 95% CI: 0.056–0.250).^16^

Furthermore, our study showed breastfeeding was associated with respiratory infections in full‐term infants (adjusted odds ratio [aOR] 0.76, 95% confidence interval [CI] 0.58–0.99)^17^ and gastrointestinal infections in late preterm infants (34–36 weeks gestation).^18^ The association with reduced hospitalization for asthma did not reach statistical significance (aOR 0.77, 95% CI 0.56–1.06).^19^ Additionally, we demonstrated that, compared with formula feeding, exclusive or partial breastfeeding was linked to lower odds of hospitalization for Kawasaki disease between 6 and 30 months of age (aOR 0.26, 95% CI 0.12–0.55) (Figure 1a).^20^

We also observed that breastfeeding was associated with achieving age‐appropriate behavior at ages 2.5 and 5.5 years (Figure 1b).^21^ These associations were most pronounced in exclusively breastfed children. For example, at age 2.5, exclusively breastfed children had lower odds of being unable to compose a two‐phrase sentence (aOR 0.43, 95% CI 0.34–0.55) or climb stairs (aOR 0.52, 95% CI 0.38–0.72). At age 5.5, they were less likely to be unable to listen without fidgeting (aOR 0.64, 95% CI 0.56–0.73).

Conversely, prolonged breastfeeding appeared to increase the risk of dental caries in early childhood (e.g., at 30 months of age, exclusive breastfeeding for at least 6–7 months was linked to an adjusted OR of 1.78, 95% CI 1.45 to 2.17, compared with exclusive formula feeding),^22^ and we observed complex relationships between breastfeeding and food allergies that varied by infantile eczema status—a topic explored in more detail in the allergy section.^23^ These findings suggest that while breastfeeding offers numerous health benefits, its relationship with certain outcomes may depend on individual risk factors and specific contexts.

#### Sleep

Early‐life exposures play critical roles in shaping children's health trajectories. Among these, sleep patterns represent a fundamental behavioral exposure that influences multiple aspects of child development. Sleep plays a critical role in growth, immunity, learning, memory consolidation, and overall well‐being. Previous studies have shown that insufficient sleep increases the risks of obesity, diabetes, cardiovascular issues, hyperactivity, aggression, and behavioral problems resembling attention‐deficit/hyperactivity disorder (ADHD).^24^, ^25^ In Japan, children tend to have shorter sleep hours and later bedtimes than in other countries, and there are concerns about the impact of this on their health and development.^26^

Using the LSN21 cohorts, our group conducted four studies examining the relationship between sleep and various health outcomes in children. Kobayashi et al. reported that irregular or late sleep schedules at age 2 were associated with increased risk of attention problems and aggressive behavior at age 8; for example, compared to an early bedtime, an irregular or late bedtime was associated with attention problems (aOR 1.62, 95% CI 1.12–2.36) and aggressiveness problems (aOR 1.81, 95% CI 1.19–2.77).^27^ Yamauchi et al. examined the association between nighttime sleep duration at age 2.5 and behavioral development at age 5.5.^28^ Children who had ≤9 h of nighttime sleep were less likely to perform age‐appropriate behavior compared with children who had ≥11 h of nighttime sleep. The adjusted odds ratios (aORs) for inability to listen without fidgeting and inability to remain patient were 1.26 (95% confidence interval [CI] 1.14–1.39) and 1.27 (95% CI 1.16–1.38), respectively. Obara et al. showed that shorter or irregular sleep at age 5.5 was linked to a higher risk of injury between ages 5.5 and 9; specifically, compared to 10–11 h of sleep, 6 h of sleep (aOR 1.40, 95% CI 1.19–1.66) and irregular sleep (aOR 1.26, 95% CI 1.10–1.43) were associated with an increased risk of injury.^29^ Uraguchi et al. reported that a dinner‐to‐bed interval of ≤120 min was associated with increased risk of gastroesophageal reflux (GER)‐related diseases compared with a longer dinner‐to‐bed time gap (>120 min), with an adjusted risk ratio (aRR) of 1.10 (95% CI 1.03–1.18) for asthma.^30^

Our group has provided valuable findings about children's sleep and various potential health outcomes. Future research should include more comprehensive sleep assessments, including naps, sleep quality, and variability. Additionally, future studies should examine the long‐term effects of sleep patterns during infancy on later development and effective intervention methods to improve various outcomes.

#### Environmental exposures

Environmental exposures constitute another critical domain that influences pediatric health. Particularly, air pollution and maternal smoking can have profound and lasting effects on children's physical and neurodevelopmental outcomes. Previous epidemiological evidence has linked passive or maternal smoking to adverse outcomes such as preterm birth^31^ and neurodevelopmental impairment,^32^ and longitudinal research has shown that children with household smoking exposure are more likely to start smoking by age 15.^33^ Furthermore, air pollution is known to be associated with low birth weight^34^ and delayed development.^35^ Building on this existing knowledge, our LSN21 study has contributed several important findings.

##### Maternal smoking

**FIGURE 2:**
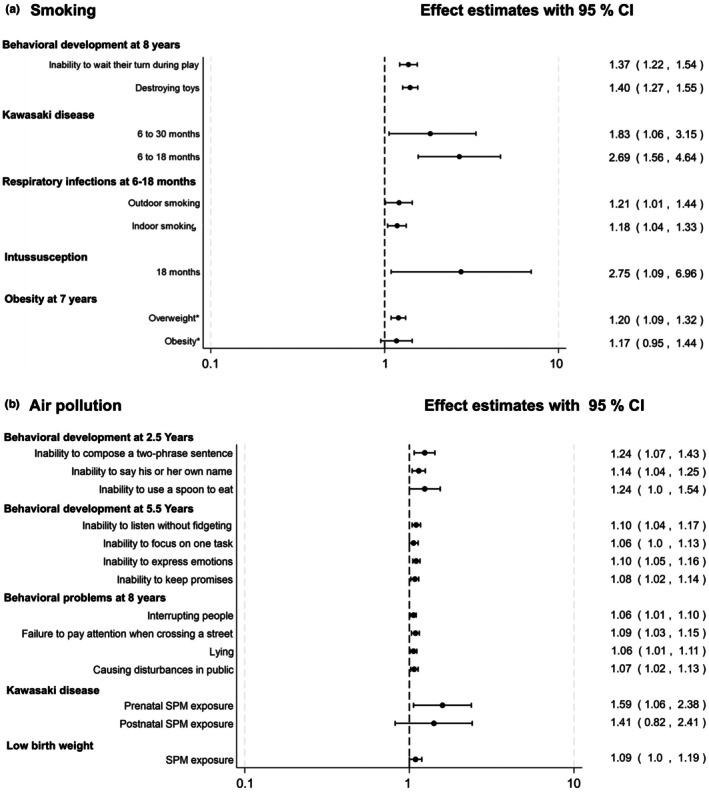
Association between environmental exposures and health outcomes in children from the LSN21 cohort. Panel A. Postnatal exposure to passive smoking, assessed by maternal smoking status at 6‐month postpartum. Panel B. Prenatal air pollution exposure, expressed as average concentrations of suspended particulate matter (SPM) and NO_2_ across the 9 months of gestation. Effect estimates are presented as odds ratios (ORs) or risk ratios (RRs) with 95% confidence intervals (CIs). Items marked with an asterisk (*) denote RRs. The *x*‐axis is presented on a logarithmic scale. All models were adjusted for relevant maternal, child, and socioeconomic characteristics. For Panel A, the reference group is children of non‐smoking mothers. Estimates correspond to the following studies from Table S1: Behavioral problems (S39), Kawasaki disease (S28), respiratory infections (S17), intussusception (S20), and obesity at age 7 (S41). For Panel B, effect estimates for behavioral development at 2.5 and 5.5 years (S10) are shown for exposure to NO_2_, while estimates for behavioral problems at 8 years (S18), Kawasaki disease (S22), and low birth weight (S7) are for exposure to SPM. The reference group is children in the lowest quartile of exposure for each respective pollutant. Panel B, the reference group is children in the lowest quartile of exposure. Estimates correspond to the following studies from Table S1: Behavioral development at 2.5 and 5.5 years (S10), behavioral problems at 8 years (S18), Kawasaki disease (S22), and low birth weight (S7).

Exposure to maternal smoking when a child was 6 months old was associated with a higher risk of child hospitalization for Kawasaki disease between 6 and 18 months of age with an aRR 2.69 (95% CI 1.56–4.64),^36^ respiratory infections (aRR 1.18, 95% CI 1.04–1.33),^37^ and intussusception before 18 months of age (aRR 2.75, 95% CI 1.09–6.96) (Figure 2a). Furthermore, maternal smoking was associated with overweight at age 7 (aRR 1.20, 95% CI 1.09–1.32)^38^ and behavioral problems at age 8. Specifically, exposure was linked to a higher risk of aggressive behaviors such as “destroying toys” (aOR 1.37, 95% CI 1.22–1.54) and attention‐deficit behaviors such as “inability to wait their turn during play” (aOR 1.40, 95% CI 1.27–1.55) (Figure 2a).^39^

##### Air pollution

The LSN21 also found that prenatal exposures to air pollution have lasting health effects. Specifically, prenatal exposures to air pollution were associated with low birth weight (e.g., for suspended particulate matter exposure, aOR 1.09, 95% CI 1.0–1.19)^40^ and hospitalization for Kawasaki disease (aOR 1.59, 95% CI 1.06–2.38) (Figure 2b). Moreover, traffic‐related air pollution during the fetal period was associated with behavioral developmental problems at ages 2.5, 5.5, and 8.^41^, ^42^ For example, at age 2.5, higher NO_2_, but not suspended particulate matter (SPM), was associated with an inability to compose a two‐phrase sentence (aOR 1.24, 95% CI 1.07–1.43), while at age 5.5, we found that the risk of behavioral difficulties (i.e., restlessness and inability to listen) increased with each quartile range in exposure to SPM among children (aOR 1.10, 95% CI 1.04–1.17) (Figure 2b).

#### Perinatal exposures

Beyond environmental factors encountered during childhood, conditions experienced during the perinatal period constitute fundamental determinants of long‐term health outcomes. The LSN21's unique linkage with vital statistics (birth record information) provides an exceptional opportunity to examine how gestational age, birth weight, and other perinatal characteristics influence subsequent development. This approach is essential for investigating developmental trajectories in vulnerable populations. Preterm birth and small‐for‐gestational‐age (SGA) status are significant risk factors for adverse developmental outcomes in children.

##### Children born preterm

**FIGURE 3:**
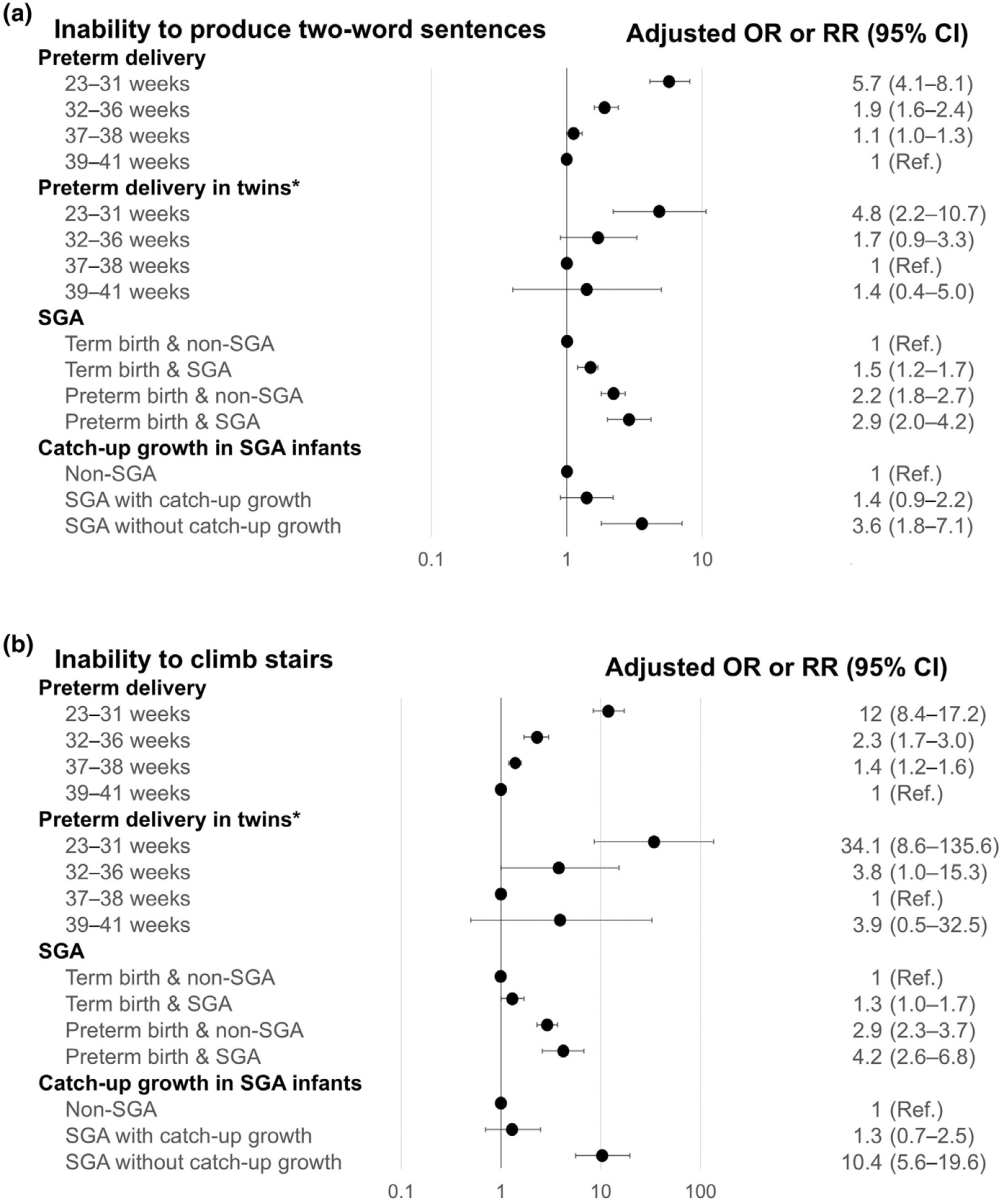
Summary of findings from the Longitudinal Survey of Newborns in the 21st Century on gestational age and developmental milestones at 2.5 years. Panels show responses to representative items from the language and motor development domains: Panel A. “Can your child compose two‐phrase sentences?” Panel B. “Can your child climb stairs?” Analyses were conducted for four subgroups: (1) the overall population, (2) twins, (3) small‐for‐gestational‐age (SGA) infants, and (4) SGA infants who achieved catch‐up growth. Effect estimates are presented as odds ratios (ORs) or risk ratios (RRs) with 95% confidence intervals (CIs). Items marked with an asterisk (*) denote RRs. The x‐axis is presented on a logarithmic scale. All models were adjusted for relevant maternal, child, and socioeconomic characteristics. The reference groups are full‐term infants (39–41 weeks) for preterm delivery comparisons, early term twins (37–38 weeks) for twin comparisons, and non‐SGA infants for SGA comparisons. Estimates correspond to the following studies from Table S1: Preterm delivery (overall population) (S3), preterm delivery in twins (S27), SGA status (S12), and catch‐up growth in SGA infants (S21).

Our analysis showed that preterm birth was associated with developmental delay at 2.5 years^43^ and behavioral problems at 8 years.^44^ Very preterm infants (<32 weeks) had an aOR of 5.7 (95% CI 4.1–8.1) for inability to produce two‐word sentences at age 2.5 years compared with full‐term infants, with incidences of this language delay being 18.9% versus 3.5%, respectively (Figure 3a).^43^ Similar patterns were observed for motor development milestones; for example, the risk for inability to climb stairs was higher in very preterm infants (aOR 12.0, 95% CI 8.4–17.2) (Figure 3b). Additionally, preterm infants experienced more frequent hospitalizations; specifically for hospitalization between 7 and 18 months, the risk was lowest for twins born at early term (37–38 weeks) but was significantly elevated for both very preterm twins (<32 weeks; adjusted RR 2.2, 95% CI 1.3–3.8) and full‐term twins (≥39 weeks; adjusted RR 1.8, 95% CI 1.0–3.2). Further analysis focusing on twins revealed a U‐shaped relationship between gestational age and both developmental delay and hospitalization, with the lowest aOR observed in those born at early term (37–38 weeks).^45^

##### Children born small‐for‐gestational‐age (SGA)

SGA status was identified as an independent risk factor for developmental delay, even among full‐term infants; for instance, term‐born SGA infants had a higher risk for inability to climb stairs compared to non‐SGA term infants (aOR 1.3, 95% CI 1.0–1.7) (Figure 3b).^46^, ^47^ Among SGA children, the absence of catch‐up growth by 2 years was associated with neurobehavioral impairment at ages 2.5, 5.5, and 8 years (Figure 3a,b).^48^, ^49^ Takeuchi et al.^48^ reported that, compared with non‐SGA infants, SGA infants who exhibited catch‐up growth by 2 years had an aOR of 0.6 (95% CI 0.19–1.89) for aggressive behavior at 8 years, whereas those who did not exhibit catch‐up growth had an aOR of 3.85 (95% CI 1.19–12.47).^48^ The incidence of aggressive behavior was 1.2% in non‐SGA infants, 0.7% in SGA infants with catch‐up growth, and 4.6% in SGA infants without catch‐up growth. The severity of SGA was associated with both hospitalization and unfavorable neurobehavioral development.^50^, ^51^, ^52^ Further research is warranted to assess the long‐term consequences of SGA birth and to develop effective interventions, particularly for those without catch‐up growth.

The preceding sections have highlighted how various early‐life exposures influence child health. Complementing this exposure‐centric approach, the following sections examine specific health outcomes of major public health significance and explore their multifactorial determinants as revealed through the LSN21 cohort studies.

### Part 2: Health outcomes and their determinants

#### Obesity and growth trajectories

Obesity is a pressing global health issue affecting millions of people worldwide.^53^, ^54^ Obesity and overweight are associated with an increased risk of mortality^55^ and childhood obesity can persist into adulthood.^56^ Multiple factors influence childhood obesity, including lifestyle factors (e.g., physical activity and diet), early‐life nutrition (including breastfeeding), and parental behavior. Lifestyle interventions combining improved diet and increased physical activity are considered the cornerstones of weight management for children.^56^ Multicomponent interventions that combine these approaches with behavioral therapy have shown effectiveness.^57^

Using data from the LSN21 cohort, we investigated several key factors associated with childhood obesity.^58^ As mentioned in the environmental exposures section, we found that maternal smoking was associated with an increased risk of childhood obesity. Additionally, our analysis of physical activity revealed that outdoor play habits at age 2.5 years were associated with lower odds of obesity at age 7 years (aOR 0.85, 95% CI 0.74–0.97).^59^ We also found that exclusive breastfeeding at 6 months was linked to lower odds of obesity at age 7 years compared with formula feeding (aOR 0.55, 95% CI 0.39–0.78).^14^ This association persisted into adolescence, with formula‐fed children having 23% higher odds of obesity at age 15 years compared with children who were exclusively breastfed (aOR 1.23, 95% CI 1.02–1.48).^15^

Regarding growth patterns, our research team tracked body mass index (BMI) and height changes from ages 1.5 to 15 years and found that children who were obese at age 15 had maintained consistently high BMI z‐scores throughout childhood, with acceleration during puberty.^60^ Early adiposity rebound was associated with subsequent overweight or obesity. Additionally, APV occurred earlier in children who became obese compared with normal‐weight children, highlighting critical developmental periods for targeted interventions.

The results of these studies from the LSN21 cohort provide meaningful insights into the associations between childhood obesity and multiple factors in Japan through a large, nationwide, long‐term, population‐based follow‐up. Future research is needed to identify additional factors associated with childhood obesity and how these factors can be modified to prevent and improve childhood obesity outcomes.

#### Allergy and respiratory infections

While obesity represents a major metabolic concern, allergic conditions and respiratory infections constitute another significant burden affecting children's quality of life and development. Lifestyle and environmental factors have been implicated in the development of allergic diseases and respiratory tract infections (RTIs), often interacting mutually, as suggested by the hygiene hypothesis.^61^ The LSN21 cohort has provided important insights into these childhood conditions with uniqueness particularly in its comprehensive assessment of diverse household environmental factors and comparison of two cohorts from different time periods (2001 and 2010).

##### Birth order and allergic diseases

Birth order emerged as a notable factor influencing the risk of asthma. Children born later showed a higher risk of developing asthma during early childhood; for example, the aRR for third‐ or later‐born children compared to first‐borns was 1.19 (95% CI, 1.05–1.35) between 30 and 42 months in the 2001 cohort. This trend reversed during school age, resulting in reduced asthma risk for later‐born children; the aRR was 0.76 (95% CI, 0.65–0.89) between 10 and 11 years in the same cohort. Interestingly, this observation remained consistent across two distinct cohorts (2001 and 2010), despite considerable differences in their demographic characteristics, suggesting robustness of the birth‐order effect.^62^, ^63^

##### Parental smoking status and allergic conditions

We examined parental smoking habits and their relationship with allergic diseases in children up to 5.5 years of age.^64^ Maternal smoking was found to significantly elevate the risk of allergic rhinitis/allergic conjunctivitis; for instance, even light maternal smoking (≦10 cigarettes/day) was associated with an increased risk (RR 1.15, 95% CI 1.02–1.30). Paternal smoking acted as an effect modifier that further amplified these risks; in households where the father also smoked, the risk associated with maternal smoking (≦10 cigarettes/day) was even higher for allergic rhinitis/allergic conjunctivitis (RR 1.21, 95% CI 1.07–1.36) and also for bronchial asthma (RR 1.33, 95% CI 1.17–1.52).

##### Breastfeeding and food allergies

Our analysis showed a nuanced relationship between breastfeeding and food allergies that was modified by infantile eczema status.^23^ Breastfeeding, particularly colostrum intake, was associated with lower odds of food allergies, but only among high‐risk children with infantile eczema (aRR 0.66, 95% CI 0.46–0.96). However, prolonged breastfeeding was associated with an increased risk of food allergies in children without infantile eczema (aRR 2.41, 95% CI 1.40–4.15).

##### Respiratory tract infections and preventive measures

We also investigated RTIs, including a comparative analysis between the 2001 and 2010 cohorts for otitis media to evaluate the impact of social changes and medical interventions. The 2010 cohort exhibited a higher prevalence of otitis media during infancy, potentially attributable to increased daycare attendance associated with dual‐income households. Nevertheless, a marked decrease in otitis media prevalence was observed from 4.5 years onward, likely reflecting the introduction of pneumococcal vaccinations in 2010.^65^ For influenza, we assessed the effectiveness of preventive behaviors and found that habitual handwashing and gargling practices were associated with a lower risk of influenza infection (aRR 0.8, 95% CI 0.8–0.9).^66^

##### Oral health and respiratory infections

We also explored the relationship between oral health and respiratory infections and found that children with dental caries had a significantly increased risk of influenza infection compared with those without dental caries (aOR 1.12, 95% CI 1.05–1.19).^67^ This association was consistent across all three age groups studied (1.5–2.5, 4.5–5.5, and 9–10 years) and persisted regardless of previous dental caries status or household income, suggesting that oral health may play a role in respiratory infection susceptibility.

These allergic diseases and RTIs significantly impact quality of life during childhood and may predispose individuals to chronic health risks into adulthood.^68^, ^69^ Despite certain limitations, our uniquely comprehensive, large‐scale birth cohort study has identified several modifiable environmental and lifestyle factors associated with these conditions that will contribute to the prevention and management strategies for these diseases.

### Part 3: Emerging research areas and future directions

The wealth of longitudinal data provided by the LSN21 continues to enable exploration beyond the established research domains presented above. As our understanding of child development evolves and new societal challenges emerge, the cohort permits investigation of previously unexplored relationships and emerging concerns. Our research has extended beyond traditional exposures to encompass child‐rearing environment, perinatal risk factors, and municipal‐level socioeconomic determinants. We have expanded our research to examine media exposure as a critical aspect of the child‐rearing environment. Our studies have revealed that early media habits influence later behavioral development,^70^ that delayed sleep in young children is associated with excessive electronic device use in later childhood,^71^ and that early television‐watching is linked to concerns about visual acuity in elementary school‐aged children.^72^

The linkage with the Perinatal Research Network database from the Japan Society of Obstetrics and Gynecology has enabled us to examine associations between perinatal factors—such as in vitro fertilization and mode of delivery—and long‐term child outcomes.^73^ These studies offer novel insights into how early medical interventions may shape developmental trajectories. Furthermore, recent investigations have focused on the potential impact of socioeconomic disparities by employing municipal‐level area deprivation indices to assess their relationship with pediatric health outcomes.^74^ Collectively, these complementary studies enhance our understanding of the multifactorial influences on child development and provide a basis for future public health strategies.

## DISCUSSION

The summary of findings from the LSN21 provides valuable insights into factors associated with child health and development in Japan. Through our narrative review of studies that were based on this nationwide birth cohort, several important patterns have emerged regarding early‐life exposures and subsequent health outcomes.

Our review demonstrates that infant feeding practices, particularly breastfeeding, exert significant influence on multiple developmental domains, including weight status, behavioral development, and infectious disease risk. The association between exclusive breastfeeding and lower obesity prevalence from childhood to adolescence suggests long‐lasting metabolic programming. However, the relationship between breastfeeding and specific outcomes is context‐dependent, as shown by the divergent associations with food allergies according to infantile eczema status. This nuanced pattern underscores the complexity of early nutritional influences and suggests that intervention strategies may need tailoring to individual risk profiles.

Sleep patterns in early childhood also emerged as important predictors of later behavioral development and injury risk. The consistent link between insufficient or irregular sleep and adverse behavioral outcomes highlights sleep hygiene as a critical, yet often under‐addressed, target for preventive efforts, particularly in Japan, where children's sleep duration is shorter than international recommendations and may therefore contribute to developmental vulnerabilities.

The LSN21 has also provided robust evidence regarding environmental exposures, particularly maternal smoking and air pollution. The wide‐ranging associations observed for these exposures—spanning respiratory health, neurodevelopment, and metabolic outcomes—underscore the importance of environmental protection policies as public health measures. Notably, the documentation of dose–response relationships between air pollution exposure and behavioral difficulties strengthens causal inference and provides compelling support for stricter environmental regulations, although residual confounding cannot be excluded.

Perinatal factors, particularly preterm birth and SGA status, remain strong determinants of developmental trajectories. The detailed characterization of developmental risks associated with these conditions allows for more targeted follow‐up strategies. Particularly noteworthy was the finding that SGA infants without catch‐up growth by age 2 showed substantially increased risk for behavioral problems at age 8, suggesting that growth monitoring during early childhood may help identify children requiring additional developmental support.

Recent LSN21‐based studies from other institutions have examined related topics and offer complementary perspectives to our review. For example, Fujiwara et al. linked higher household income and a smaller number of siblings to increased Kawasaki disease risk through age 10,^4^ suggesting a hygiene‐related socioeconomic gradient. A Kyoto University group reported that early daycare attendance mitigated asthma and allergic rhino‐conjunctivitis up to age 9, whereas severe infant bronchiolitis remained a strong predictor of later asthma.^5^ Using oral health items, Aida et al. documented widening absolute inequalities in caries treatment between ages 2.5 and 5.5 years.^6^ Finally, Nagayoshi et al. showed that greater paternal housework participation at 6 months was associated with a 23% reduction in the odds of mothers' frequent spanking at 3.5 years.^75^ These external findings highlight social gradients, childcare contexts, and paternal roles not covered in our 59‐paper overview, which underscores the breadth of insights the LSN21 resource can generate.

The methodological strengths of the LSN21 deserve acknowledgment. Its large, nationally distributed sample, high initial response rates, and long follow‐up enhance generalizability to the broader Japanese child population. Additionally, the parallel cohorts from 2001 and 2010 create unique opportunities to evaluate secular trends and policy impacts. For instance, the observed reduction in otitis media prevalence from age 4.5 years in the 2010 cohort compared to the 2001 cohort provides suggestive evidence for the effectiveness of pneumococcal vaccination introduced in 2010.

Nonetheless, several methodological limitations warrant consideration. The reliance on parental self‐report for most outcome measures introduces potential reporting bias, particularly for behavioral assessments in which parental perceptions may be influenced by expectations and social desirability. Despite comprehensive adjustment for socioeconomic indicators and developmental factors, residual confounding cannot be excluded, given the observational design. Additionally, attrition—although moderate—may be selective, with families who are experiencing greater socioeconomic disadvantage or child developmental difficulties being more likely to discontinue participation.

Beyond these methodological considerations, the LSN21 has several conceptual and design limitations that constrain its ability to address certain research questions. Notably, the absence of biological samples precludes investigation of genetic factors, epigenetic mechanisms, or biomarkers of environmental exposure; complementary cohort studies in Japan provide important additional perspectives. The Japan Environment and Children's Study (JECS), a nationwide birth cohort initiated in 2011, collects extensive biological specimens including maternal blood, cord blood, breast milk, and children's urine and blood samples, enabling detailed assessments of environmental exposures and biological response pathways.^76^, ^77^ Similarly, the Tohoku Medical Megabank Birth and Three‐Generation Cohort Study (TMM BirThree Cohort Study) offers multigenerational data, including genetic information, which allows researchers to explore questions about intergenerational patterns of health outcomes through its own distinct research program.^78^, ^79^ While these cohorts each employ distinct designs that preclude direct data integration, they provide complementary perspectives that enrich understanding of child development in Japan. The LSN21's strength in long‐term, nationally representative follow‐up with detailed social and behavioral assessments is complemented by the JECS's focus on environmental exposures and biological mechanisms, and Tohoku Medical Megabank's emphasis on genetic and intergenerational factors.

The demonstrated importance of early‐life exposures underscores the need for preventive measures beginning in pregnancy and infancy. Specifically, promoting breastfeeding, supporting healthy sleep habits, reducing environmental pollutant exposure, and eliminating secondhand smoke, particularly in households with young children, represent evidence‐based priorities for child health promotion. The identified vulnerabilities of preterm and SGA infants, especially those without catch‐up growth, suggest the need for enhanced developmental surveillance and support services for these high‐risk groups.

As participants in the cohort transition into adulthood, future research directions include examination of associations between early exposures and adult health outcomes, including cardiometabolic risk factors, mental health, and social functioning. Additionally, integration of LSN21 data with advanced analytic approaches such as machine learning may help reveal complex patterns and interactions not apparent through traditional statistical methods. Finally, international comparative studies among the LSN21 and similar cohorts in other countries could elucidate how cultural and social contexts modify developmental processes and the impact of early‐life exposures.

The Longitudinal Survey of Newborns in the 21st Century has generated substantial evidence regarding factors that shape child health and development in contemporary Japan. The findings summarized in this review offer an empirical foundation for developing effective interventions and policies to promote optimal developmental outcomes. As Japan continues to address the demographic challenges of population aging and declining birth rates, ensuring the health and well‐being of each child becomes increasingly vital. The LSN21 provides critical scientific evidence to guide these efforts, while also contributing to the global understanding of child development in diverse social contexts. Furthermore, a future systematic or integrative review that incorporates the growing body of LSN21‐based research from all institutions would be a valuable next step to synthesize the full spectrum of the cohort's contributions to science.

## AUTHOR CONTRIBUTIONS

Drs. Takashi Yorifuji and Naomi Matsumoto jointly conceived and conceptualized the narrative review and determined its overall structure. Dr. Matsumoto drafted the full manuscript and harmonized the tone and style across all subsections. Drs. Rumi Matsuo, Yuka Yamamura, Takahiro Tsuge, Tomoka Kadowaki, Kensuke Uraguchi, Kei Tamai, Kazue Nakamura, and Akihito Takeuchi each prepared the first draft of their assigned subsection. Dr. Yorifuji critically reviewed and revised the manuscript, providing intellectual input and giving final approval. All authors have read and approved the final version of the manuscript.

## FUNDING INFORMATION

This study was supported by JSPS KAKENHI Grant Number JP23K16329 and JP24K13507. The JSPS had no role in the design and conduct of the study.

## CONFLICT OF INTEREST STATEMENT

T. Yorifuji received the Academic Award 2020 of the Japan Pediatric Society in recognition of the LSN21 research program. The Award carried no financial grant for the present work and did not influence the study design, data interpretation, or manuscript preparation. All other authors declare no conflicts of interest.

## Supporting information


Table S1

## Data Availability

The data that support the findings of this study are available from the Ministry, Health, Labour, and Welfare in Japan. Restrictions apply to the availability of these data, which were used under license for this study. Data are available with the permission of the Ministry, Health, Labour, and Welfare in Japan.
