# Supplementary material for: Early‐life exposures and child health outcomes: A narrative review of LSN21 research in Japan
Source: Pediatr Int. 2025 Nov 10;67(1):e70258. doi: 10.1111/ped.70258 (PMC12603348; doi:10.1111/ped.70258)
Supplement: Supplementary file 1 — Table S1 [file PED-67-e70258-s001.docx]

| Supplementary Table 1. Comprehensive list of the 59 peer-reviewed publications derived from the Longitudinal Survey of Newborns in the 21st Century (2013 – 2025). | | | | | |  |
| --- | --- | --- | --- | --- | --- | --- |
| ID(S#) | Authors | Title | Journal | Exposure domain | Outcome domain |  |
| S1 | Kato et al. | Association of Birth Length and Risk of Hospitalisation among Full-term Babies in Japan. | Paediatr Perinat Epidemiol. | Perinatal | Other Outcomes |  |
| S2 | Yamakawa et al. | Breastfeeding and obesity among schoolchildren: a nationwide longitudinal survey in Japan. | JAMA Pediatr. | Infant feeding | Obesity/Growth Trajectory |  |
| S3 | Kato et al. | Associations of preterm births with child health and development: Japanese population-based study. | J Pediatr. | Perinatal | Neuro-behavioral |  |
| S4 | Yorifuji et al. | Breastfeeding and Behavioral Development: A Nationwide Longitudinal Survey in Japan. | J Pediatr. | Infant feeding | Neuro-behavioral |  |
| S5 | Kobayashi et al. | Poor toddler-age sleep schedules predict school-age behavioral disorders in a longitudinal survey. | Brain Dev. | Sleep | Neuro-behavioral |  |
| S6 | Yamakawa et al. | Breastfeeding and Hospitalization for Asthma in Early Childhood: A Nationwide Longitudinal Survey in Japan | Public Health Nutr. | Infant feeding | Allergic/Respiratory |  |
| S7 | Yorifuji　et al. | Outdoor Air Pollution and Term Low Birth Weight in Japan | Environ Int. | Environmental | Other Outcomes |  |
| S8 | Yamakawa et al. | Long-term effects of breastfeeding on children's hospitalization for respiratory tract infections and diarrhea in early childhood in Japan. | Matern Child Health J | Infant feeding | Allergic/Respiratory |  |
| S9 | Kato et al. | Association of breast feeding with early childhood dental caries: Japanese population-based study | BMJ Open. | Infant feeding | Other Outcomes |  |
| S10 | Yorifuji　et al. | Prenatal Exposure to Traffic-Related Air Pollution and Child Behavioral Development Milestone Delays in Japan. | Epidemiology. | Environmental | Neuro-behavioral |  |
| S11 | Higa Diez M et al. | Preterm birth and behavioural outcomes at 8?years of age: a nationwide survey in Japan | Arch Dis Child. | Perinatal | Neuro-behavioral |  |
| S12 | Takeuchi et al. | Neurodevelopment in full-term small for gestational age infants: A nationwide Japanese population-based study. | Brain Dev. | Perinatal | Neuro-behavioral |  |
| S13 | Nosaka et al. | Development of a Japanese scale for assessment of paediatric normal weight | Resuscitation. | Other exposures | Other Outcomes |  |
| S14 | Yorifuji et al. | Breastfeeding and Risk of Kawasaki Disease: A Nationwide Longitudinal Survey in Japan. | Pediatrics. | Infant feeding | Allergic/Respiratory |  |
| S15 | Inoue et al. | Children's Media Use and Self-Regulation Behavior: Longitudinal Associations in a Nationwide Japanese Study | Matern Child Health J | Environmental | Neuro-behavioral |  |
| S16 | Takeuchi et al. | Behavioral Outcomes of School-aged Full-term Small-for-gestational-age Infants: a Nationwide Japanese Population-based Study | Brain Dev. | Perinatal | Neuro-behavioral |  |
| S17 | Yamakawa et al. | Maternal Smoking Location at Home and Hospitalization for Respiratory Tract Infections among Children in Japan. | Arch Environ Occup Health. | Environmental | Allergic/Respiratory |  |
| S18 | Yorifuji et al. | Prenatal exposure to outdoor air pollution and child behavioral problems at school age in Japan. | Environ Int. | Environmental | Neuro-behavioral |  |
| S19 | Nosaka et al. | New age-based weight estimation formulae for Japanese children. | Pediatr Int. | Perinatal | Other Outcomes |  |
| S20 | Nakahara et al. | Maternal smoking as a risk factor for childhood intussusception. | Arch Environ Occup Health | Perinatal | Other Outcomes |  |
| S21 | Takeuchi et al. | Catch-up Growth and Neurobehavioral Development among Full Term, Small-for-gestational-age Children: a Nationwide Japanese Population-based Study | J Pediatr. | Perinatal | Neuro-behavioral |  |
| S22 | Yorifuji　et al. | Intrauterine and Early Postnatal Exposure to Particulate Air Pollution and Kawasaki Disease: A Nationwide Longitudinal Survey in Japan | J Pediatr. | Environmental | Allergic/Respiratory |  |
| S23 | Kikkawa et al. | Birth Order and Pediatric Allergic Disease: A Nationwide Longitudinal Survey | Clin Exp Allergy. | Other exposures | Allergic/Respiratory |  |
| S24 | Kato et al. | National data showed that delayed sleep in six-year-old children was associated with excessive use of electronic devices at 12 years | Acta Paediatr. | Sleep | Neuro-behavioral |  |
| S25 | Tokinobu et al. | Association of early daycare attendance with allergic disorders in children: a longitudinal national survey in Japan. | Arch Environ Occup Health. | Environmental | Allergic/Respiratory |  |
| S26 | Yoshimoto et al. | Population-based longitudinal study showed that children born small for gestational age faced a higher risk of hospitalisation during early childhood | Acta Paediatr. | Perinatal | Other Outcomes |  |
| S27 | Tamai K et al. | Associations of gestational age with child health and neurodevelopment among twins: A nationwide Japanese population-based study. | Early Hum Dev. | Perinatal | Neuro-behavioral |  |
| S28 | Yorifuji et al. | Early Childhood Exposure to Maternal Smoking and Kawasaki Disease: A Longitudinal Survey in Japan. | Sci Total Environ. | Environmental | Allergic/Respiratory |  |
| S29 | Takeuchi et al. | Catch-up growth and behavioral development among preterm, small-for-gestational-age children: a nationwide Japanese population-based study. | Brain Dev | Perinatal | Neuro-behavioral |  |
| S30 | Matsumoto et al. | Breastfeeding and risk of food allergy: A nationwide birth cohort in Japan. | Allergol Int. | Infant feeding | Allergic/Respiratory |  |
| S31 | Nakamura et al. | Exclusively Breastfeeding Modifies the Adverse Association of Late Preterm Birth and Gastrointestinal Infection: A Nationwide Birth Cohort Study | Breastfeed Med. | Infant feeding | Other Outcomes |  |
| S32 | Tamai et al. | Associations of Birth Weight for Gestational Age with Child Health and Neurodevelopment among Term Infants: A?Nationwide Japanese Population-Based Study | J Pediatr | Perinatal | Neuro-behavioral |  |
| S33 | Matsuo et al. | Television-watching in the early years of life and the association with parents' concerns about decreased visual acuity in their elementary school-aged child: results of a nationwide population-based longitudinal survey of Japan | Jpn J Ophthalmol | Other exposures | Neuro-behavioral |  |
| S34 | Matsumoto et al. | Association between Dental Caries and Influenza Infection in Children: A Japanese Nationwide Population-Based Study. | Children (Basel) | Other exposures | Allergic/Respiratory |  |
| S35 | Matsumoto et al. | Trajectory of body mass index and height changes from childhood to adolescence: a nationwide birth cohort in Japan. | Sci Rep. | Perinatal | Obesity/Growth Trajectory |  |
| S36 | Obara et al. | Short or Irregular Sleep Duration in Early Childhood Increases Risk of Injury for Primary School-Age Children: A Nationwide Longitudinal Birth Cohort in Japan. | Int J Environ Res Public Health. | Sleep | Other Outcomes |  |
| S37 | Takeuchi et al. | Preterm birth and Kawasaki disease: a nationwide Japanese population-based study. | Pediatr Res. | Perinatal | Allergic/Respiratory |  |
| S38 | Tamai et al. | Sports participation and preterm birth: a nationwide birth cohort in Japan. | Pediatr Res. | Perinatal | Other Outcomes |  |
| S39 | Ariyoshi et al. | Early childhood exposure to maternal smoking and behavioral development. | Arch Environ Occup Health. | Environmental | Neuro-behavioral |  |
| S40 | Yamauchi et al. | Association of nighttime sleep with behaviors in Japanese early childhood. | Pediatr Int. | Sleep | Neuro-behavioral |  |
| S41 | Yamashita et al. | Early childhood exposure to maternal smoking and obesity: A nationwide longitudinal survey in Japan. | Clin Obes. | Environmental | Obesity/Growth Trajectory |  |
| S42 | Kadowaki et al. | Breastfeeding at 6?months of age had a positive impact on overweight and obesity in Japanese adolescents at 15?years of age. | Acta Paediatr. | Infant feeding | Obesity/Growth Trajectory |  |
| S43 | Higuchi Y et al. | Association between infant breastfeeding practices and timing of peak height velocity: A nationwide longitudinal survey in Japan. | Pediatr Res. | Infant feeding | Obesity/Growth Trajectory |  |
| S44 | Uraguchi K et al. | Association between handwashing and gargling education for children and prevention of respiratory tract infections: a longitudinal Japanese children population-based study. | Eur J Pediatr. | Environmental | Allergic/Respiratory |  |
| S45 | Namba T et al. | Evaluation of the association of birth order and group childcare attendance with Kawasaki disease using data from a nationwide longitudinal survey. | Front Pediatr. | Other exposures | Allergic/Respiratory |  |
| S46 | Ohyama A et al. | A nationwide birth cohort in Japan showed increased risk of early childhood hospitalisation in infants born small for gestational age. | Acta Paediatr. | Perinatal | Other Outcomes |  |
| S47 | Uraguchi K et al. | Association Between Clinical Remission of Infantile-Onset Allergic Rhinitis During the School-Age Period and the Type of Housing: A Longitudinal Population-Based Japanese Study. | J Pediatr Health Care | Environmental | Allergic/Respiratory |  |
| S48 | Takanaga S et al. | Small for Gestational Age at Birth and Risk of Kawasaki Disease: A Nationwide Birth Cohort in Japan | Acta Medica Okayama | Perinatal | Allergic/Respiratory |  |
| S49 | Uraguchi K et al. | Pediatric otitis media in Japan: A nationwide longitudinal study of the pre- and post-pneumococcal conjugate vaccine eras born in 2001 and 2010. | Vaccine | Other exposures | Allergic/Respiratory |  |
| S50 | Murata et al., | Childcare and Child Development in Japan. | Acta Medica Okayama | Other exposures | Neuro-behavioral |  |
| S51 | Matsumoto N et al. | In vitro fertilization and long-term child health and development: nationwide birth cohort study in Japan | Eur J Pediatr. | Perinatal | Allergic/Respiratory |  |
| S52 | Hiraoka et al., | A nationwide longitudinal survey of infantile injury and its recurrence in Japan. | Sci Rep. | Perinatal | Other Outcomes |  |
| S53 | Uraguchi K et al. | Association Between Dinner-to-Bed Time and Gastroesophageal Reflux-Related Diseases in Children. | Cureus. | Sleep | Allergic/Respiratory |  |
| S54 | Kobayashi M et al. | Impact of Birth Order on Paediatric Allergic Diseases: A National Birth Cohort in Japan | Clin Exp Allergy. | Other exposures | Allergic/Respiratory |  |
| S55 | Matsumoto N et al. | Cesarean delivery on child health and development in Japanese nationwide birth cohort. | Sci Rep. | Perinatal | Allergic/Respiratory |  |
| S56 | Shigehara K et al. | Maternal smoking during infancy increases the risk of allergic diseases in children: a nationwide longitudinal survey in Japan. | Allergy Asthma Clin Immunol. | Environmental | Allergic/Respiratory |  |
| S57 | Tsuge et al., | Outdoor playing during preschool was associated with a reduced risk of school-age obesity in Japan | Acta Paediatrica | Environmental | Obesity/Growth Trajectory |  |
| S58 | Matsumoto N et al. | Area Deprivation and Health Outcomes in Preschool Children in Japan: A Nationwide Cohort Study | Journal of Epidemiology | Other exposures | Allergic/Respiratory |  |
| S59 | Yabuuchi T et al. | Changes in body mass index during early childhood and school-age asthma prevalence classified by phenotypes and sex | Pediatrics International | Perinatal | Allergic/Respiratory |  |
| Entries are presented in chronological order and assigned unique identifiers S1 – S59. The table details each study’s first author, publication year, exposure domain, child-health outcome category, and full citation. These S-numbers are referenced in the main text (e.g., Table 1) but are independent of the numerical reference citations used in the manuscript’s reference list. | | | | | |  |
|  |  |  |  |  |  |  |
